# Hertz-rate metropolitan quantum teleportation

**DOI:** 10.1038/s41377-023-01158-7

**Published:** 2023-05-10

**Authors:** Si Shen, Chenzhi Yuan, Zichang Zhang, Hao Yu, Ruiming Zhang, Chuanrong Yang, Hao Li, Zhen Wang, You Wang, Guangwei Deng, Haizhi Song, Lixing You, Yunru Fan, Guangcan Guo, Qiang Zhou

**Affiliations:** 1 Institute of Fundamental and Frontier Sciences, University of Electronic Science and Technology of China, Chengdu, 610054 China; 2 Shanghai Institute of Microsystem and Information Technology, Chinese Academy of Sciences, Shanghai, 200050 China; 3 Southwest Institute of Technical Physics, Chengdu, 610041 China; 4 CAS Key Laboratory of Quantum Information, University of Science and Technology of China, Hefei, 230026 China

**Keywords:** Quantum optics, Single photons and quantum effects

## Abstract

Quantum teleportation can transfer an unknown quantum state between distant quantum nodes, which holds great promise in enabling large-scale quantum networks. To advance the full potential of quantum teleportation, quantum states must be faithfully transferred at a high rate over long distance. Despite recent impressive advances, a high-rate quantum teleportation system across metropolitan fiber networks is extremely desired. Here, we demonstrate a quantum teleportation system which transfers quantum states carried by independent photons at a rate of 7.1 ± 0.4 Hz over 64-km-long fiber channel. An average single-photon fidelity of ≥90.6 ± 2.6% is achieved, which exceeds the maximum fidelity of 2/3 in classical regime. Our result marks an important milestone towards quantum networks and opens the door to exploring quantum entanglement based informatic applications for the future quantum internet.

## Abstract (web summary)

Quantum teleportation through metropolitan distance, on the campus of the University of Electronic Science and Technology of China - Quantum Networks No. 1 of UESTC, achieves a rate of 7.1 ± 0.4 Hz.

## Graphical abstract

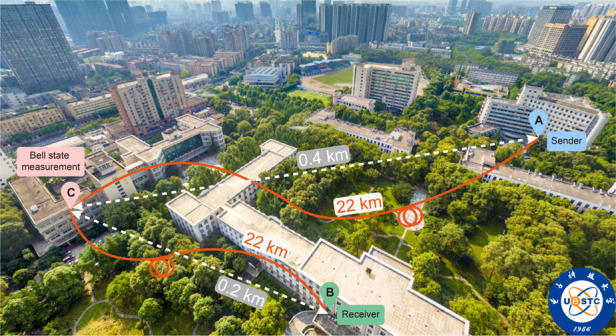

## Introduction

Quantum teleportation^1^ enables the ‘disembodied’ transfer of an unknown quantum state to a remote location by using quantum entanglement resource with the help of quantum measurement and classical communication. It lies at the heart of the realization of quantum information technologies such as quantum network^2–4^ and distributed quantum computation^5^. Since its initial proposal by Bennett et al. in 1993^1^, quantum teleportation has been demonstrated in various platforms, including atomic ensembles^6^, single atoms^7^, trapped ions^8,9^, solid-state quantum systems^10^, nuclear magnetic resonance^11^ and quantum optics^12–33^. Teleportation systems based on quantum optics offer a promising avenue towards quantum networks, which can be realized in continuous-variable (CV) and discrete-variable (DV) systems, respectively. For instance, the transfer and retrieval for both coherent states^28–31^ and nonclassical states^32^ have been experimentally realized with optical modes in CV systems, providing a method to realize deterministic quantum teleportation. However the distance of CV system is limited to around ten kilometers^30,31^, due to the possible increased fragility with respect to the losses of quantum channels^34^. For global-scale quantum networks^2,3^, the distribution range of quantum states needs to be greatly extended to thousands of kilometers using quantum teleportation in DV systems. Till now, this has been realized with multiple degrees of freedom over several meters to more than one thousand kilometers, from the table-top experiments^12–17^ to real-world demonstrations^18–26^. Especially, by using a low-Earth orbit Micius satellite^35^, quantum teleportation over 1200 km has been achieved^25,26^. Despite impressive results, a high-rate quantum teleportation system has yet to be demonstrated, which is desired for advancing the development of quantum networks.

Here we report an experimental realization of a Hertz-rate quantum teleportation system through fiber over a metropolitan range. Our demonstration relies on a high-performance time-bin entangled quantum light source with a single piece of fiber-pigtailed periodically poled lithium niobate (PPLN) waveguide. The quantum states to be teleported are carried by a weak coherent single-photon source with decoy states. The indistinguishability of photons after prior quantum states distribution through fiber channels is ensured with a fully running feedback system. As an important feature of our demonstration, photonic time-bin qubits are teleported at a rate of 7.1 ± 0.4 Hz over a 64-km-long fiber channel. An average single-photon fidelity of ≥90.6 ± 2.6% is achieved with the decoy state method. Our implementation establishes an important milestone towards quantum internet.

## Results

### Experimental setup

**Fig. 1:**
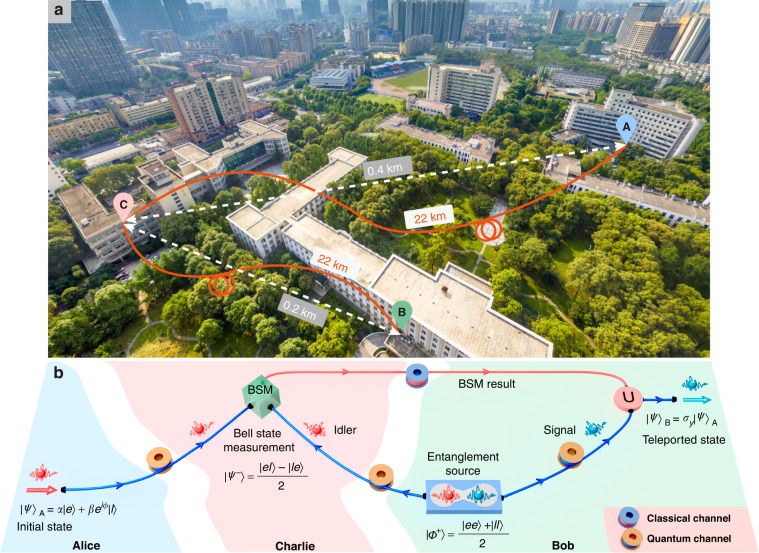
Three-node quantum teleportation system. **a** Aerial view of the teleportation system. Alice ‘A’ is located at network’s switching room, Bob ‘B’ and Charlie ‘C’ are located at two separated laboratories. All fibers connecting the three nodes belong to the UESTC backbone network. During the experiment, only the signals created by Alice, Bob and Charlie are transferred through these ‘dark’ fibers. **b** Scheme of the teleportation system. Alice prepares the initial state ${|\psi {{\rangle }}}_{{\rm{A}}}$ with a weak coherent single-photon source and sends it to Charlie through a quantum channel ($\text{Q}{\text{C}}_{\text{A}\to \text{C}}$). An entanglement source at Bob generates a pair of entangled photons in the state $|{\varPhi }^{+}{{\rangle }}$ and then sends the idler photon to Charlie via another quantum channel ($\text{Q}{\text{C}}_{\text{B}\to \text{C}}$). The signal photon is stored in a fiber spool. Charlie implements a joint Bell-state measurement (BSM) between the qubit sent by Alice and Bob, projecting them onto one of the four Bell states $|{\psi }^{-}{{\rangle }}$. Then the BSM result is sent to Bob via a classical channel (CC), who performs a unitary (U) transformation on the signal photon ${|\psi {{\rangle }}}_{{\rm{B}}}$ to recover the initial state ${|\psi {{\rangle }}}_{{\rm{A}}}$ (see Materials and Methods)

**Fig. 2:**
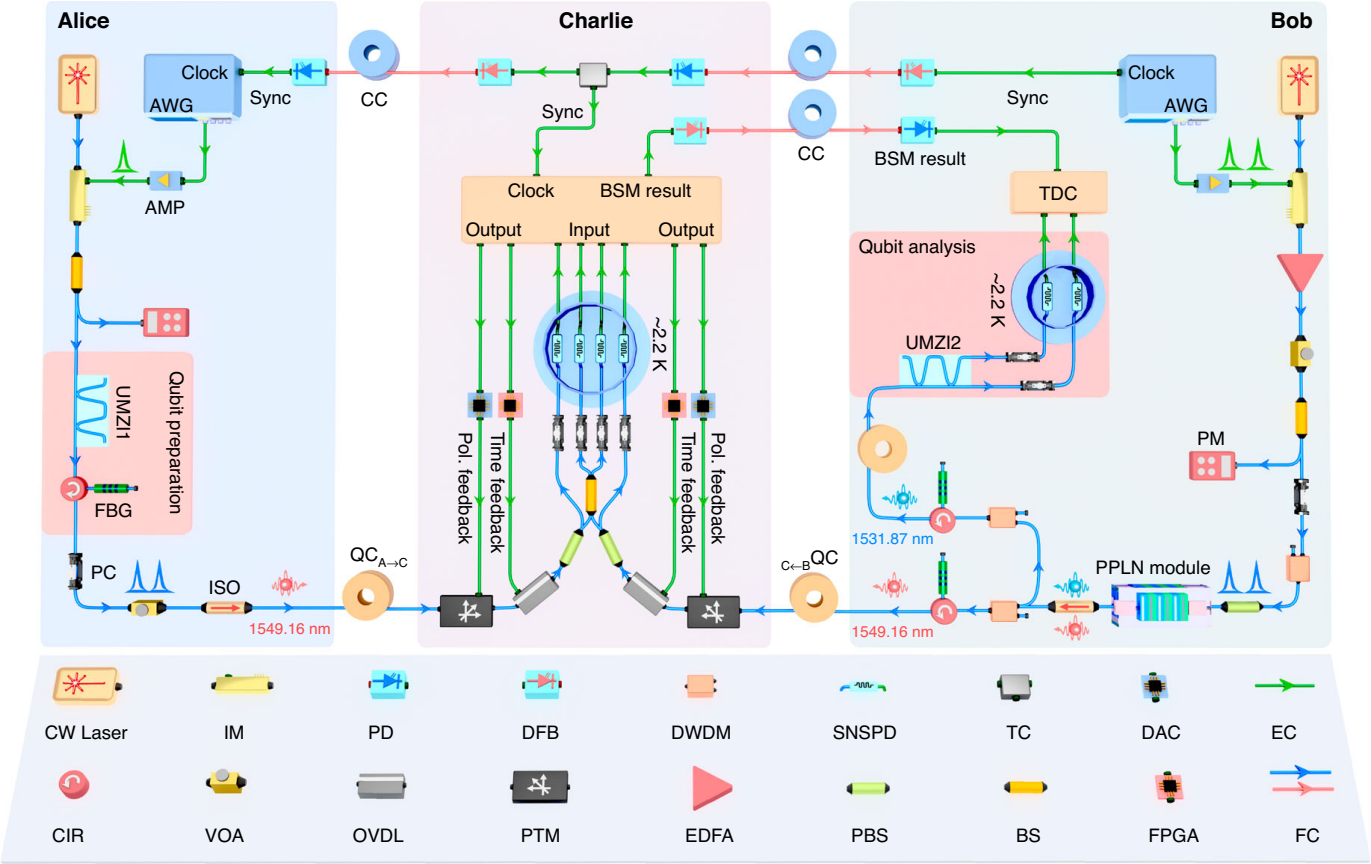
Experimental setup. Alice’s setup. The 65-ps-long pulses of light are created by modulating 1549.16 nm continuous wave (CW) laser (PPCL300, PURE Photonics) at 500 MHz rate with an intensity modulator (IM). The drive signal is generated by an arbitrary waveform generator (AWG) and amplified by a 25-GHz-bandwidth amplifier (AMP), synchronized with Bob’s clock through a classical channel (CC, red line). A fiber beam splitter (BS) with a ratio of 99:1 and a powermeter (PM) are used to monitor the power of the laser pulses. Subsequently, an unbalanced Mach–Zehnder interferometer (UMZI1, MINT, Kylia) with a path-length difference equivalent to 625 ps is applied to prepare the time-bin qubits to be teleported. Following with a spectrally filtering by a 10-GHz-wide fiber Bragg grating (FBG) combined with an optical circulator (CIR) and a strong attenuation to the single photon level by a variable optical attenuator (VOA), the prepared qubits are sent to Charlie through a 22 km fiber quantum channel (QC), $\text{Q}{\text{C}}_{\text{A}\to \text{C}}$ blue line - featuring 6.8 dB loss. Bob’s setup. Two pump laser pulses separated by 625 ps with the same repetition rate of Alice are generated using a 1540.56 nm CW laser (PPCL300, PURE Photonics) in conjunction with an IM. The pump power is amplified, adjusted, and monitored by an erbium-doped fiber amplifier (EDFA), VOA, and 99:1 BS with a PM, respectively. A polarization controller (PC) and polarization beam splitter (PBS) are used to ensure the polarization alignment for maximizing the efficiency of phase matching in the periodically poled lithium niobate waveguide. The time-bin entangled state of $|{\varPhi }^{+}{{\rangle }}={2}^{-1/2}({|ee}{{\rangle }}+{|ll}{{\rangle }})$ is generated using cascaded second-order nonlinear processes in the PPLN waveguide module (see Materials and Methods), with mean photon pair number of ${\mu }_{\text{SPDC}}=0.042$ in the experiment. The entangled photon pairs are spectrally filtered into signal (1531.87 nm) and idler (1549.16 nm) ones using dense-wavelength division multiplexers (DWDMs) and FBGs with a full width at half maximum bandwidth of 125 GHz and 10 GHz, respectively. The idler photons are sent to Charlie via another 22 km fiber QC, $\text{Q}{\text{C}}_{\text{B}\to \text{C}}$ - featuring 6.4 dB loss and the state of signal photons (stored in a 20 km fiber spool) is analyzed using UMZI2 (625 ps transmission delay, MINT, Kylia), two superconducting nanowire single photon detectors (SNSPDs, P-CS-16, PHOTEC) - cooled to 2.2 K in a cryostat and with 80% detection efficiency, and a time-to-digital converter (TDC, ID900, ID Quantique). Charlie’s setup. The photons from Alice and Bob are projected onto the $|{\psi }^{-}{{\rangle }}$ Bell state using a 50:50 BS and two SNSPDs with 60% detection efficiency. To ensure the indistinguishability of the two photons distributed through a 22-km-long fiber channel for each, we actively stabilize the arrival times and polarization with an active and automatic feedback system on both $\text{Q}{\text{C}}_{\text{A}\to \text{C}}$ and $\text{Q}{\text{C}}_{\text{B}\to \text{C}}$ channels. The timing and polarization feedback signals (Time feedback and Pol. feedback) are generated from field-programmable gate array (FPGA) circuits and digital to analog convertor (DAC) circuits, respectively, and sent to optical variable delay lines (OVDLs, MDL-002, General Photonics) and polarization tracker modules (PTMs, POS-002, General Photonics) to compensate for the time and polarization drifts. Two optical isolators (ISOs) with ~55 dB isolations are used to shied Alice and Bob from attacks. The synchronization (Sync) between the three nodes is performed by classical optical pulses through classical channels (CCs), and assisted with AWGs, distributed feedback (DFB) lasers, photon detectors (PDs) and a tee connector (TC). Both QC and CC are dark fiber cables (FC). The electronic cables (EC) are denoted by green lines (see Materials and Methods for more details about stabilization and synchronization)

Figure 1a shows an aerial photography of the campus of University of Electronic Science and Technology of China (UESTC) indicating the distances between the locations Alice, Bob and Charlie. Figure 1b shows the scheme of our teleportation system, and Fig. 2 depicts its experimental setup. To be compatible with the structure of quantum networks^36^, the quantum state to be teleported should be carried by an independent single-photon source, which is different with the Rome scheme^13^. In our demonstration, the quantum bit (qubit) sender, Alice, located at a switching room of the backbone network on the campus, prepares a weak coherent single-photon source, which is used to encode photonic time-bin qubit, i.e., a single-photon wavepacket in a coherent superposition of two time bins. The time-bin qubit is obtained by passing the single-photon wavepacket through an unbalanced Mach–Zehnder interferometer (UMZI) with path-length difference ∆*τ*. The time-bin qubit can be written as ${|\psi {{\rangle }}}_{{\rm{A}}}=\alpha {|e}{{\rangle }}+\beta {e}^{i\phi }{|l}{{\rangle }}$, where ${|e}{{\rangle }}$ represents the early time bin (i.e., a photon having passed through the short arm of the interferometer); ${|l}{{\rangle }}$ is the late time bin (i.e., a photon having passed through the long arm); *ϕ* is a relative phase between ${|e}{{\rangle }}$ and ${|l}{{\rangle }}$, and ${\alpha }^{2}+{\beta }^{2}=1$. Alice sends the created quantum states carried by single-photon wavepackets to Charlie, located at a laboratory at a flight distance of 400 m away, through a quantum channel (QC) of 22 km, i.e., $\text{Q}{\text{C}}_{\text{A}\to \text{C}}$, including 2 km deployed fiber in field and 20 km fiber spool. The quantum information receiver, Bob, located at another laboratory, 210 m from Charlie, shares with Charlie a pair of time-bin entangled photonic qubits in the state of $|{\varPhi }^{+}{{\rangle }}={2}^{-1/2}({|ee}{{\rangle }}+{|ll}{{\rangle }})$, with one at 1549.16 nm (idler) and the other at 1531.87 nm (signal). The idler photons are distributed through another 22 km QC to Charlie, i.e., $\text{Q}{\text{C}}_{\text{B}\to \text{C}}$, including 2 km deployed fiber in field and 20 km fiber spool. Charlie performs the joint Bell-state measurement (BSM) between the qubits sent by Alice and Bob, using a 50:50 fiber beam splitter (BS). We select only projections onto the singlet state of $|{\psi }^{-}{{\rangle }}={2}^{-1/2}({|el}{{\rangle }}-{|le}{{\rangle }})$, which can be realized by the detection of one photon in each output port of BS with a time difference of 625 ps. When a $|{\psi }^{-}{{\rangle }}$ has been successfully detected, the BSM result is sent to Bob over a classical channel (CC) by means of an optical pulse. In this case, the signal photons at Bob (stored in a 20-km-long fiber spool) are projected onto the state of ${|\psi {{\rangle }}}_{{\rm{B}}}={\sigma }_{y}{|\psi {{\rangle }}}_{{\rm{A}}}$, with *σ*_*y*_ being a Pauli matrix. The synchronization of the teleportation system is made through the CCs (see Materials and Methods). All fiber spools used in our system are non-zero dispersion-shifted single-mode optical fiber (G.655, Yangtze Optical Fibre and Cable).

### Prior entanglement distribution

**Fig. 3:**
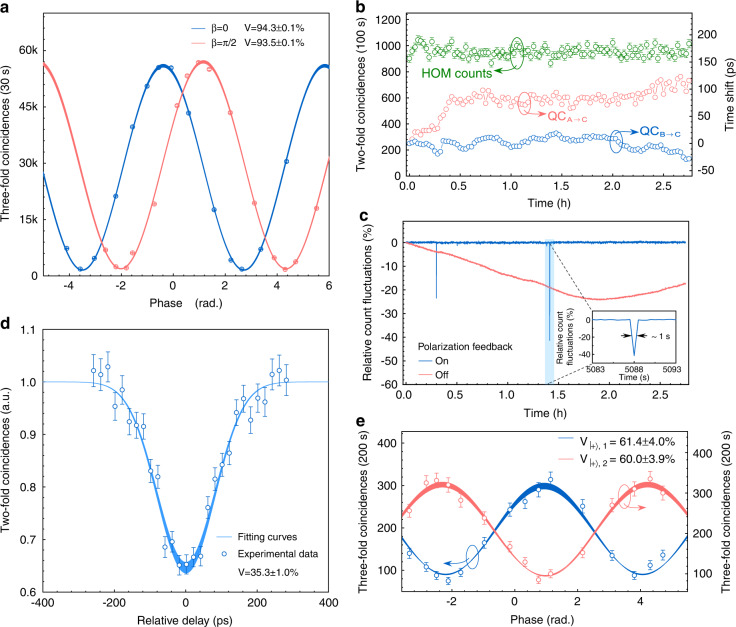
Experimental results of prior entanglement distribution, indistinguishability of photons at Charlie, and teleportation of equatorial states. **a** Two-photon Franson interference fringes of time-bin entanglement source after distribution. Blue and red circles show the coincidence counts for the phase of UMZI1 on idler path set at 0 and $\pi /2$, respectively. The visibilities of the fitting curves are 94.3 ± 0.1% and 93.5 ± 0.1%, with the uncertainties calculated using the Monte Carlo method. **b** Automatic timing control on $\text{Q}{\text{C}}_{\text{A}\to \text{C}}$ and $\text{Q}{\text{C}}_{\text{B}\to \text{C}}$, respectively. Red (blue) circles represent the drifts of Alice’s (Bob’s) photons arrival times with respect to the system clock. Green circles represent the coincidence counts of HOM interference per 100 s with active feedback. **c** Automatic polarization feedback on $\text{Q}{\text{C}}_{\text{A}\to \text{C}}$. Red (Blue) lines correspond to relative fluctuations of $\text{Q}{\text{C}}_{\text{A}\to \text{C}}$ with feedback off (on). **d** Normalized HOM interference curve after fiber transmission of photons at Charlie. The visibility of the HOM curve is 35.3 ± 1.0%, corresponding to a single-photon indistinguishability of 88.8 ± 2.4% at Charlie, with details shown in Supplementary Note S2. **e** Teleportation of equatorial states. The red and blue circles represent the three-fold coincidence counts from the two outputs of UMZI2 at Bob. The visibilities of the fitting curves are 61.4 ± 4.0% and 60.0 ± 3.9%, respectively, indicating the coherence property of Alice’s state is successfully teleported to the signal photons. All error bars are calculated by Monte Carlo simulation assuming Poissonian detection statistics

The property of prior entanglement distribution is measured before performing the BSM of quantum teleportation. In the experiment, we distribute the idler photons through $\text{Q}{\text{C}}_{\text{B}\to \text{C}}$ to Charlie while the signal photons are held by a 20 km spool of fiber at Bob. The distributed time-bin entanglement property is characterized with the Franson interferometer, with details shown in Supplementary Note S1. The visibilities of two-photon interference fringes are 94.3 ± 0.1% and 93.5 ± 0.1%, respectively, as shown in Fig. 3a. The error bars of visibilities are calculated by Monte Carlo simulation assuming Poissonian detection statistics. This result indicates that the quantum entanglement property still maintains after being distributed over 42 km fiber channels. Furthermore, it also allows us to ensure that parameters of two UMZIs can be remotely set as the same in our setup, which is a crucial requirement for the quantum teleportation processes.

### Indistinguishability of photons at Charlie

Alice’s and Bob’s photons need to be indistinguishable at Charlie for a successful BSM, which is difficult in long distance quantum teleportation. The spatial and spectral indistinguishabilities are ensured by using single-mode fibers and identical fiber Bragg grating (FBG) filters for both photons. The path-length difference and polarization of the photons are stabilized with an active and automatic feedback system (see Materials and Methods). The experimental results of indistinguishabilities at Charlie are shown in Fig. 3b, c. With our fully running feedback system, we measure the Hong-Ou-Mandel (HOM) interference curve^37^ with the time-bin qubits from Alice and Bob, respectively. The result given in Fig. 3d shows a HOM-dip with a visibility of 35.3 ± 1.0% by Gaussian fitting, approaching the upper bound of 40% between the coherent state and the thermal state, which corresponds to a single-photon indistinguishability of 88.8 ± 2.4% at Charlie, with details shown in Supplementary Note S2.

### Quantum teleportation results

Two classes of quantum states are prepared to be teleported from Alice to Bob: one class contains qubits lying on the equator of the Poincare sphere (coherent superpositions of ${|e}{{\rangle }}$ and ${|l}{{\rangle }}$ with equal amplitudes, ${|\psi {{\rangle }}}_{{\rm{A}}}={2}^{-1/2}({|e}{{\rangle }}+{e}^{i\phi }{|l}{{\rangle }})$, and the other class contains the two poles of the Poincare sphere (${|e}{{\rangle }}$ and ${|l}{{\rangle }}$). For the equatorial states, a successful teleportation implies Bob’s photon to be in a superposition state (${|\psi {{\rangle }}}_{{\rm{B}}}={\sigma }_{y}{|\psi {{\rangle }}}_{{\rm{A}}}$). Conditional on the successful BSM result from Charlie through CC, we observe sinusoidal curves of three-fold coincidence with visibilities of 61.4 ± 4.0% and 60.0 ± 3.9% for two outputs of UMZI2, respectively, as shown in Fig. 3e. The maximum value of three-fold coincidence counts is 335 ± 18 for 200 s, indicating that a quantum teleportation rate of 7.1 ± 0.4 Hz is achieved excluding an extra measurement loss of 6.25 dB, i.e., 5.30 dB from UMZI2 and 0.95 dB from single-photon detection. With the measured visibilities, the fidelity for the equatorial states can be calculated as ${F}_{\text{equator}}=\left(1+V\right)/2$, corresponding to a fidelity of 80.4 ± 2.0% for the equatorial states^15^, which alone can already represent a strong indication of the quantum teleportation. It is worth mentioning that all the visibilities are obtained without subtracting the background noise. With the UMZI1 in Alice removed, we directly prepare ${|e}{{\rangle }}$ (${|l}{{\rangle }}$) state with a single temporal mode and send it to Charlie for BSM. For the measurement, Bob removes the UMZI2 and accumulates three-fold coincidence counts at the corresponding time bins within a coincidence window of 200 ps. The fidelity ${F}_{e/l}$ can be calculated by ${F}_{e/l}={R}_{\text{c}}/\left({R}_{\text{c}}+{R}_{\text{w}}\right)$, where *R*_c_ and *R*_w_ represent the probability of detecting the correct and wrong state in the pole basis, respectively. The measured fidelity for the ${|e}{{\rangle }}$ input state is 92.2 ± 1.0% and for the ${|l}{{\rangle }}$ input state 92.4 ± 1.1%. Assuming that the performance of equatorial states is the same, i.e., ${F}_{+}={F}_{-}={F}_{+i}={F}_{-i}={F}_{\text{equator}}$, we apply ${F}_{\text{avg}}=\left(4{F}_{\text{equator}}+{F}_{e}+{F}_{l}\right)/6$ to obtain an average fidelity of 84.3 ± 1.7%, which is significantly above the maximum fidelity of 2/3 in classical regime.

**Fig. 4:**
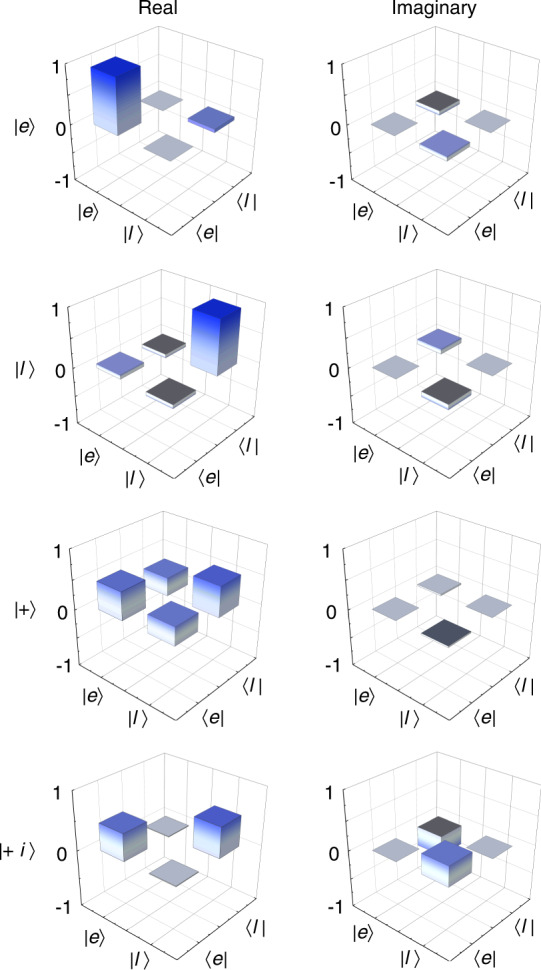
Density matrices of four quantum states after teleportation. The real and imaginary parts of the reconstructed density matrices of four different input states prepared at Alice. The state labels denote the states expected after teleportation. The mean photon number per qubit is ${\mu }_{\text{A}}=0.029$ and the mean photon pair number is ${\mu }_{\text{SPDC}}=0.042$

**Fig. 5:**
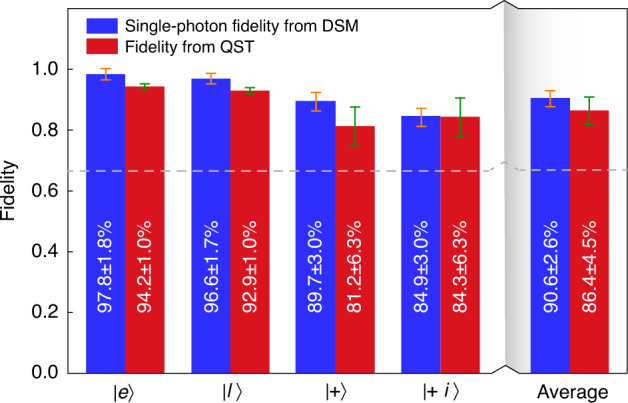
Individual and average fidelities of four teleported states with ideal state, obtained with quantum state tomography (QST) method and the decoy state method (DSM). Red bars are fidelities measured using QST. Blue bars are fidelities obtained with DSM. Both fidelities from the two methods exceed the classical limit of 2/3, i.e., the dashed gray line. For the QST and DSM we set ${\mu }_{\text{SPDC}}=0.042$. Error bars are calculated using Monte Carlo simulation, assuming Poissonian detection statistics (see Supplementary Note S5 and Supplementary Tables S3 and S4 for more calculation and statistics details)

Furthermore, we reconstruct the density matrices *ρ* of the quantum states after teleportation using quantum state tomography (QST) method^38^, as described in Supplementary Note S3. Four well-defined states (${|e}{{\rangle }}$, ${|l}{{\rangle }}$, $|+\rangle$ and $|+i\rangle$, $|+{{\rangle }}={2}^{-1/2}({|e}{{\rangle }}+{|l}{{\rangle }})$ and $|+i{{\rangle }}={2}^{-1/2}({|e}{{\rangle }}+{i|l}{{\rangle }})$ are created to perform QST in our system. We calculate fidelities of the quantum teleportation by $F={ }_{\mathrm{B}}\langle\psi|\rho| \psi\rangle_{\mathrm{B}}$ with the expected states (${|\psi {{\rangle }}}_{{\rm{B}}}$). Figure 4 shows the density matrices of four quantum states after teleportation obtained by QST. The fidelities for all four prepared states are given in Fig. 5, which exceed the maximum classical value of 2/3. The more decoherence of $|+{{\rangle }}$ and $|+i{{\rangle }}$ state results from the residual distinguishability of the photons (see Supplementary Note S2), which will not cause any effect on ${|e}{{\rangle }}$ and ${|l}{{\rangle }}$ states. This can be improved by further eliminating the distinguishability of photons in all degrees of freedom, i.e., spatial, spectral, temporal, and polarization degrees^39^. The uncertainty of teleportation fidelities is calculated assuming Poissonian detection statistics and using Monte Carlo simulation. The average fidelity ${F}_{\text{avg}}=\left(2\left({F}_{+}+{F}_{+i}\right)+{F}_{e}+{F}_{l}\right)/6$ is 86.4 ± 4.5%, showing the quantum nature of the disembodied state transfer from Alice to Bob.

It is noted that the classical fidelity bound of 2/3 is only applied when Alice’s initial states carried with genuine single photons, rather than weak coherent states prepared with attenuated laser pulses. Here we utilize the decoy state method (DSM)^40–42^ to estimate the performance of our system given that genuine single photons are used^24^. In the experiment, we prepare quantum states${|e}{{\rangle }}$, ${|l}{{\rangle }}$, $|+\rangle$ and $|+i{{\rangle }}$ with varying the mean photon number per qubit at Alice among three values (${\mu }_{\text{A}}^{\text{s}}=0.088$, ${\mu }_{\text{A}}^{\text{d}}=0.029$ and ${\mu }_{\text{A}}^{\text{v}}=0$, where ${\mu }_{\text{A}}^{\text{s}}$, ${\mu }_{\text{A}}^{\text{d}}$ and ${\mu }_{\text{A}}^{\text{v}}$ are the mean photon numbers of the signal, decoy and vacuum state, respectively) and perform quantum teleportation, with details shown in Supplementary Tables S3 and S4. Based on these results, we calculate the lower bounds of ${F}_{e/l}^{1}$ and ${F}_{+/+i}^{1}$ as shown in Fig. 5, with ${F}_{\text{avg}}^{1}$ ≥ 90.6 ± 2.6%, which significantly violates the classical bound of 2/3 by more than 9 standard deviations, clearly demonstrating the capability of our system for high-fidelity teleportation. We present an analytical model of our teleportation system^24^, and observe a good quantitative agreement between theory and experiment (see Supplementary Notes S4 and S5). Finally, we conclude the key metrics of our teleportation system in Table 1, where the state-of-the-art teleportation systems in DV with photonic qubits sent by an independent source are summarized as a comparison. Note that, in Table 1, the state-transfer distance corresponds to the total length of quantum channel between Alice and Bob, while the teleportation distance is defined as the bee-line spatial separation between the location of the BSM station and the signal photon at the time of the BSM projection^24^.

**Table 1:** Comparison between the state-of-the-art results and our work

Year	State-transfer distance (km)	Teleportation distance (km)	Fidelity	Rate (Hz)	Channel	Ref.
1997	0.1	0.1	77%	3*10^−2^	Free space	^12^
2003	2	0.1	81%	5*10^−2^	Fiber	^14^
2004	6	0.1	78%	5*10^−2^	Fiber	^15^
2007	1	0.6	93%	2*10^−3^	Fiber	^19^
2012	143	0.1	86%	3*10^−2^	Free space	^21^
2012	97	0.1	80%	8*10^−2^	Free space	^22^
2014	25	0.1	81%	2*10^−3^	Fiber	^33^
2015	102	0.1	84%	2*10^−2^	Fiber	^16^
2016	60	6	91%	5*10^−4^	Fiber	^23^
2016	17	6	80%	2*10^−1^	Fiber	^24^
2017	1127	0.1	80%	4*10^−2^	Free space	^25^
2020	44	0.1	89%	9*10^−3^	Fiber	^17^
**2022**	**64**	**0.2**	**91%**	**7.1** **±** **0.4**	**Fiber**	**Our work**

## Discussion

Metrics for a quantum network are of course the rate, fidelity and distance of quantum teleportation. Although our work has moved one important step closer to high-speed quantum teleportation over a metropolitan area, further increases of teleportation rate in our system could be reached by increasing the repetition rate of system, the efficiencies of SNSPDs and BSM, and using multiple spectral channels^43^. Further insights into photonic quantum information encoding, the use of multiple degrees of freedom^44^ or multiple qubits^45^ will also certainly increase the information capacity of quantum teleportation system based on hyperentanglement Bell-state analysis^46,47^. The deviations of the fidelity from unity in our system are mostly due to multiphoton events of quantum light sources and the remaining distinguishability of the two photons undergoing the BSM. We may replace the SNSPDs with photon-number resolving SNSPDs^48,49^ to allow post-selection of multiphoton events. Alternatively, another promising solution to multiphoton events from Alice is applying single quantum emitters that can generate individual photons deterministically^50^. Further, the indistinguishability between the photons from Alice and Bob could be improved by using narrower FBGs (see Supplementary Note S2). To extend the teleportation distance, the combination of low-Earth-orbit satellite links^25,26^ and quantum repeater architecture^51,52^ may provide a prospective avenue for the long distances beyond 5000 km or so^53^. It is also noted that the signal photons in our system, centered at 1531.87 nm, both in terms of wavelength and spectral width, are compatible with quantum memory in erbium-doped materials^54–56^. This, in conjunction with entanglement swapping, constitutes an elementary link of a quantum network, which has been realized recently between two solid states quantum memories^57–59^.

In conclusion, we have demonstrated a quantum teleportation system over metropolitan area, where a 7.1 ± 0.4 Hz teleportation rate is achieved with up to 64 km state-transfer distance. An average fidelity of 86.4 ± 4.5% is measured using QST. Using the DSM, we obtain an average single-photon fidelity of ≥90.6 ± 2.6%. Our results are further supported by an analytical model which is consistent with measurements of the quantum teleportation system. Finally, our work establishes the possibility of the high-speed quantum information transmission, which serves as a blueprint for the construction of metropolitan quantum network and eventually towards the global quantum internet.

## Materials and methods

### PPLN module design

The entangled photon pairs are generated using cascaded nonlinear processes of second harmonic generation (SHG) and spontaneous parameter down conversion (SPDC) in a periodically poled lithium niobate (PPLN) waveguide module^43,60,61^. By fiber-integrating the PPLN waveguide with noise-rejecting filters^61^, the spontaneous Raman scattering noise photons generated in the module are greatly reduced, and entangled photon pairs with a high rate under the same coincidence-to-accidental ratio (CAR) are obtained. More details about parameters of our PPLN module are listed in Supplementary Table S1.

### Synchronization

The master clock of the teleportation system is generated from an arbitrary waveform generator (AWG) at Bob and converted into optical pulses by using distributed feedback (DFB) lasers. The optical pulses are sent through the classical channels (CCs) from Bob to Charlie, and from Charlie to Alice. Charlie and Alice receive the optical pulses and convert them into electrical signals using photon detectors (PDs), the outputs of which are used for synchronization at both stations. As shown in Fig. 2, Charlie is connected to Alice and Bob through dark fibers of campus backbone networks. Among them, the fiber that carries photonic qubits is referred to as the quantum channel (QC) and the fiber that transmits optical pulses is referred to as the CC. In addition, both unbalanced interferometers at Alice and Bob are calibrated and stabilized by single photon interference. This method permits us to carry out the preparation and measurement of time-bin qubits with stable phases.

### Stabilization to ensure the indistinguishability of photon

A successful Bell-state measurement (BSM) relies on the indistinguishability of the two photons, which are generated by independent sources and have been distributed through a 22-km-long fiber channel for each. To do that, the spatial indistinguishability is ensured by using single-mode optical fibers. The spectral indistinguishability of photons from Alice and Bob is ensured by spectral filtering with separate 10-GHz-wide temperature-stabilized fiber Bragg gratings (FBGs). However, in a real-world quantum teleportation system, the length and birefringence of the optical fiber are influenced by external environments, such as the strain and temperature fluctuations, which make photons distinguishable in degrees of temporal mode and polarization mode. To overcome these challenges, we develop the following experimental techniques to stabilize the QCs (see Supplementary Fig. S4 for the schematic).

#### Automatic timing control

We measure the arrival times of Alice’s and Bob’s photon respectively, and compensate their time drifts with respect to the system clock. As shown in Fig. 2, the detection signals of photons from the reflection port of the polarization beam splitter (PBS) and the system clock are sent to a time-to-digital converter (TDC) to record the arrival times of photons from each channel for every 10 seconds. The drift signals of arrival time in each channel are obtained with a field-programmable gate array (FPGA) circuit. Then the drift signals are fed to two optical variable delay lines (OVDLs, MDL-002, General Photonics) in the two QCs to compensate for the arrival time drifts with a resolution of 1 ps. As shown in Fig. 3b, within ~3 h measurement, a time shift of 120 ps (−40 ps) is applied to compensate timing drift in timing through $\text{Q}{\text{C}}_{\text{A}\to \text{C}}$ ($\text{Q}{\text{C}}_{\text{B}\to \text{C}}$), respectively. During the measurement, the minimum count of the HOM interference is 954 ± 37 per 100 s. The result shows that despite the timing drifts in two QCs are much larger than the duration of the single-photon wavepacket, the teleportation should still succeed with our active timing control.

#### Automatic polarization control

At Charlie, the photons from Alice and Bob pass through two PBSs so that the polarization indistinguishability between them is naturally satisfied. However, to ensure the minimum loss of photons through PBS, the polarization must be set and maintained, which can be achieved by an automatic polarization control system to compensate for the polarization drifts. In our experiment, we perform automatic polarization control on both $\text{Q}{\text{C}}_{\text{A}\to \text{C}}$ and $\text{Q}{\text{C}}_{\text{B}\to \text{C}}$, with schematic setup shown in Supplementary Fig. S4. For instance, to control the polarization of $\text{Q}{\text{C}}_{\text{A}\to \text{C}}$, we monitor the detection counts of Alice’s photons from the reflection port of the PBS per 10 seconds. The count number is sent to a digital to analog convertor (DAC) circuit, which generates analog feedback signal. The feedback signal is fed to a polarization track module (PTM, POS-002, General Photonics), which ensures the maximum counts of the transmission port of the PBS by automatic polarization control. As shown in Fig. 3c, the average fluctuations of $\text{Q}{\text{C}}_{\text{A}\to \text{C}}$ within ~3 h are limited to 0.2% with our automatic polarization feedback (blue line), and to 15.4% without feedback (red line). The vibrations in the blue line are caused by actively controlling the polarization state of the photons, which recovers within 1 second with our polarization feedback system, as shown in the inset of Fig. 3c.

### Data acquisition

Charlie performs $|{\psi }^{-}{{\rangle }}$ BSM with photonic qubits to be teleported from Alice and idler photons (1549.16 nm) from Bob. When two photons arrive on two different detectors with a time delay of 625 ps, a successful $|{\psi }^{-}{{\rangle }}$ detection is obtained. Successful BSM results are transmitted through the CC to Bob by classical optical pulses, which are converted back to electrical signals by using a PD. The signals are then sent to a TDC to perform a three-fold coincidence measurement with detections of stored signal photons (1531.87 nm) from the outputs of UMZI2 at Bob. The time delay between the BSM result and the detection of signal photons is implemented by using a configurable electronic delay module on the TDC.

### Supplementary information

Supplementary Information

## Data Availability

All data needed to evaluate the conclusions in the paper are present in the paper and/or the Supplementary Information. Additional data related to this paper may be requested from the authors.
