# Author Correction: Hertz-rate metropolitan quantum teleportation

**DOI:** 10.1038/s41377-024-01442-0

**Published:** 2024-08-28

**Authors:** Si Shen, Chenzhi Yuan, Zichang Zhang, Hao Yu, Ruiming Zhang, Chuanrong Yang, Hao Li, Zhen Wang, You Wang, Guangwei Deng, Haizhi Song, Lixing You, Yunru Fan, Guangcan Guo, Qiang Zhou

**Affiliations:** 1 Institute of Fundamental and Frontier Sciences, University of Electronic Science and Technology of China, Chengdu, 610054 China; 2 Shanghai Institute of Microsystem and Information Technology, Chinese Academy of Sciences, Shanghai, 200050 China; 3 Southwest Institute of Technical Physics, Chengdu, 610041 China; 4 CAS Key Laboratory of Quantum Information, University of Science and Technology of China, Hefei, 230026 China

**Keywords:** Quantum optics, Single photons and quantum effects

Correction to: *Light: Science & Applications*

10.1038/s41377-023-01158-7 published online 10 May 2023

After the publication of the original article^1^, it was brought to the author attention that the information on Ref. 12 in Table 1 was incorrect. The incorrect and correct information is shown in this correction article, all other cell values remain correct.

**Incorrect information ref. 12**

**Table:** 

Year	State-transfer distance (km)	Teleportation distance (km)	Fidelity	Rate (Hz)	Channel	Ref.
1997	0.1	0.1	70%	3*10^−2^	Fiber	^12^

**Correct information ref. 12**

**Table:** 

Year	State-transfer distance (km)	Teleportation distance (km)	Fidelity	Rate (Hz)	Channel	Ref.
1997	0.1	0.1	77%	3*10^−2^	Free space	^12^

The original paper has been updated.
