# Supplementary material for: Hertz-rate metropolitan quantum teleportation
Source: Light Sci Appl. 2023 May 10;12:115. doi: 10.1038/s41377-023-01158-7 (PMC10172182; doi:10.1038/s41377-023-01158-7)
Supplement: Supplementary file 1 — Supplementary Information [file 41377_2023_1158_MOESM1_ESM.docx]

**Supplementary Information for**

**Hertz-rate metropolitan quantum teleportation**

Si Shen,^1^ Chenzhi Yuan,^1^ Zichang Zhang,^1^ Hao Yu,^1^ Ruiming Zhang,^1^ Chuanrong Yang,^1^ Hao Li,^2^ Zhen Wang,^2^ You Wang,^1,3^ Guangwei Deng,^1,4^ Haizhi Song,^1,3^ Lixing You,^2^ Yunru Fan,^1^ Guangcan Guo,^1,4^ Qiang Zhou^1,4^,*

*^1^Institute of Fundamental and Frontier Sciences, University of Electronic Science and Technology of China, Chengdu 610054, China.*

*^2^Shanghai Institute of Microsystem and Information Technology, Chinese Academy of Sciences, Shanghai 200050, China.*

*^3^Southwest Institute of Technical Physics, Chengdu 610041, China.*

*^4^CAS Key Laboratory of Quantum Information, University of Science and Technology of China, Hefei 230026, China.*

*Corresponding author. Email: zhouqiang@uestc.edu.cn (QZ)

**Note S1: Generation and prior distribution of** **entangled photon pairs**

Figure S1 shows the setup for the generation and characterization of entangled photon pairs. In the experiment, the signal (idler) photon counts () and the coincidence (accidental coincidence) counts () are measured under different pump power levels, which can be expressed as^1-3^:

where *R* represents the generation rate of photon pairs in periodically poled lithium niobate (PPLN) waveguide, () is the collection efficiency of the signal (idler) wavelength, () is the spontaneous Raman scattering (SpRS) noise photons generated in PPLN module, () is the dark counts of superconducting nanowire single photon detectors (SNSPDs) in signal (idler) channel, is the width of the coincidence window - 200 ps in our experiment. The single-side counts of the signal and idler photons are measured under different pump power levels - black circles in Fig. S2 (a and b). The green lines are quadratic polynomial fitting curves, and the quadratic (red line) and linear (blue line) components present the contribution of entangled photon pairs and noise photons, respectively. We can extract the quadratic terms from the fitting curves of . Then the generation rate of photon pairs *R* can be calculated via Eq. with the measured and under each pump power level. The average number of entangled photon pairs () at different pump power levels is obtained by , where is the repetition rate of entangled photon pairs, i.e., 500 MHz in our experiment. For all measurements in the teleportation experiment, the pump power is set at 6.12 mW, giving a = 0.042. Figure S2 (c and d) show the and measured with different pump power, respectively.

Figure S3 shows the schematic for prior entanglement distribution in our experiment. The length of the fiber spool is about 20 km. In both Charlie and Bob, two unbalanced Mach-Zehnder interferometers (UMZIs, UMZI1 at Charlie and UMZI2 at Bob) and superconducting nanowire single photon detectors (SNSPDs) are used to project the time-bin qubit onto the time or energy bases, where the time delay difference (625 ps) between the long and short arms of UMZIs equals to the interval of time-bins^4^. Charlie sends the detection signal of 1549.16 nm photons to Bob through the classical channel (CC), who performs three-fold coincidence counts with the system clock and detection signal of 1531.87 nm photons. We obtain two-photon interference fringes when the phase of UMZI1 at Charlie is fixed at 0 and and of UMZI2 at Bob is scanned, as shown in Fig. 3a in the main text.

**Note S2:** **Indistinguishability of photons at Charlie**

We apply the Hong-Ou-Mandel (HOM) interference to estimate the single-photon indistinguishability of single-photon wavepackets at Charlie. Let us consider a 50:50 beam splitter (BS) with two input ports and two output ports, with field operators for input and output ports represented as , and , , respectively. We can express these four field operators as:

The two-photon coincidence probability *P* with unit indistinguishability can be expressed^5,6^:

where and are the mean photon number for the two input fields of and , respectively. and are second order auto-correlation values for two input fields of and , respectively. represents the interference term, whose value depends on the overlap between the two input fields. When two input fields are completely overlapped with , the interference term = 0 corresponds to the dip of HOM curve. When two input fields are temporally separated, the interference term = 1 corresponds to the wing of HOM curve.

Assuming the input fields and are ideal thermal and coherent field, respectively, = 2 and = 1 are obtained. We can derive the visibility of HOM interference from Eq. :

Supposing the mean photon number for the two input fields are identical with , a theoretical upper bound of HOM interference visibility = 40% is obtained. The HOM visibility measured in our experiment is = 35.3±1.0% with identical mean photon number from Alice and Bob (see Fig. 3d in the main text), which indicates that the single-photon indistinguishability of single-photon wavepacket at Charlie is = 88.8±2.4%, i.e., a residual distinguishability of 11.2±2.4%. The measured second order auto-correlation value of the idler photons (1549.16 nm) after 10 GHz spectral filtering is 1.88±0.04. Thus we argue that the reduction in the indistinguishability from the ideal value of 1 could be attributed to the imperfection of spectral shape of the filter, which can be further improved.

By using Eq. , we calculate the predicted HOM visibility as a function of the mean photon number of teleported qubits , shown by the blue line in Fig. S5a. The red circles in Fig. S5a shows the experimental HOM visibility versus , which agrees well with the calculation results. For all the calculations and measurements, the mean photon pair number is 0.042.

**Note S3: Tomography of teleported state**

We use quantum state tomography (QST) to reconstruct the density matrix of the quantum state after teleportation, and then calculate the quantum teleportation fidelity. Based on the scheme in Ref.^7^, the density matrix of a single time-bin qubit can be represented by Stokes parameters:

where, is the Pauli matrix and represents the Stokes parameter. Stokes parameters can be calculated by projecting the states to the basis of , , , , and , where , , and are represented by a linear combination of and :

The projection measurement counts are , , , , and , respectively. Using these counts, we obtain the Stokes parameters as follows:

Substituting Eq. into Eq. , the density matrix after teleportation can be obtained. The teleportation fidelity is calculated with the expected state by:

**Note S4: Analytical model of teleportation system**

We apply the analytical model in Ref.^8^ to figure out the main parameters in our experiment, thus improving the performance of our teleportation system. In this method, the sum of the fidelity and the error rate is equal to 1. Hence, the fidelity of the teleported state can be predicted with the probability of three-fold coincidence counts for successful teleportation () and that for failure teleportation ()

Notice that in the following experiments, all the measured fidelities are calculated by using Eq. , with obtained by the maximum three-fold coincidence counts and obtained by the minimum three-fold coincidence counts.

Since the average photon number per qubit in our system is much less than 1, we ignore the contribution of higher-order terms to the predicted results. Here, we only consider the following cases: and ( and denote the number of photons arriving at the BS from Alice and Bob. is the signal photon number generated by entangled photon pairs). The three-fold coincidence counts probability per qubit for different cases can be expressed by:

where represents the average entangled photon pair number per qubit , is the average photon number per qubit at Alice, is the transmission probability of quantum channel from Alice to Charlie (), represent the transmission probability of quantum channel from Bob to Charlie (), is the transmission probability of signal photons (stored in a fiber spool), is the detection efficiency of SNSPDs used for BSM, is the detection efficiency of SNSPDs for signal photons. All of the experimental parameters in the teleportation system are listed in Table S2.

The teleportation fidelity of input states on the equator of the Bloch sphere is given by:

where represents the degree of indistinguishability between photons from Alice and Bob (Note S2). The teleportation rate can be expressed as:

where represents the repetition rate of teleportation system.

From Eqs. and , we predict the fidelity of state as a function of - the blue curve in Fig. S5b. An increase of the fidelity with is observed. The fidelity reaches a maximum value when the probabilities of receiving one photon from Alice and Bob are equal at Charlie^9^. With further increasing of , the multiphoton events decrease the fidelity. The teleportation rate predicted by our model as a function of state-transfer distance is shown in Fig. S5c. The teleportation rate decays exponentially as the state-transfer distance increases with the length of fiber spool at Bob. Figure S5d shows the predicted fidelity of state with different state-transfer distances, which remains unchanged as the distance increases. As shown in Fig. S5e, we plot the fidelity of state as a function of with = 0.029. The fidelity decreases with due to the multiphoton events from the entangled photon pairs. To verify the prediction of our model, we carry out experiments under different situations. Red circles in Fig. S5b show the measured fidelities of state with different . The fidelities of state and teleportation rates with different state-transfer distances of 44, 64 and 84 km are illustrated by red circles in Fig. S5 (c and d), respectively. The measured fidelities of states with different are shown by the red circles in Fig. S5e. From the above results, we observe an excellent quantitative agreement between experiments and model.

**Note S5: Predicting the fidelity of genuine single photon with decoy state method**

The decoy state method (DSM) is originally put forward to defend against photon number splitting attack in quantum key distribution by preparing sender’s source with multiple intensity levels^10-12^. We apply this method and follow Ref.^8^ to extract the fidelity and rate of single photon in our teleportation system. In the experiment, the teleported states at Alice are prepared by attenuated laser pulses with different average photon numbers (denoted as signal state , decoy state , and vacuum state ). The error rate for the single photon component of the weak coherent single-photon source is upper bounded by ^12^

and are the error rates when Alice’s teleported state is encoded in the decoy state and vacuum state, respectively. is the corresponding gain, i.e., the probability for three-fold coincidence counts when a weak coherent state with mean photon number is prepared at Alice. is the yield for a vacuum state, and is the lower bound of yield for the genuine single photon state. From Eq. , we can estimate the upper bound of the error rate of the teleported state prepared using genuine single photons. The parameters except in Eq. can be measured experimentally. Assuming a weak coherent state is created at Alice with a mean photon number of , the corresponding gain can be expressed as:

where n is the photon number of the quantum state, is the yield of an n-photon state, which cannot be measured directly from the experiment, with an exception of . From Ref.^13^, can be calculated by:

where is the gain of the signal state. With measured , , , , , and , the upper bound of the error rate can be obtained from Eqs. and . Therefore, we can calculate the lower bound of the teleportation fidelity for quantum states using a genuine single photon source:

Based on these results, we obtain an average single-photon fidelity of ≥ 90.6±2.6%, with gains and fidelities shown in Tables S3 and S4, respectively. Considering only the term in Eq. , the corresponding gain contributed by a single photon input is obtained by , resulting in a single-photon teleportation rate of ≥ 6.1±0.7 Hz in our system.

Furthermore, we change the average entangled photon pair number () and measure the fidelities of state with DSM. The green circles in Fig. S5e show the fidelities of state remain unchanged with increasing (see Tables S5 and S6 for more details).


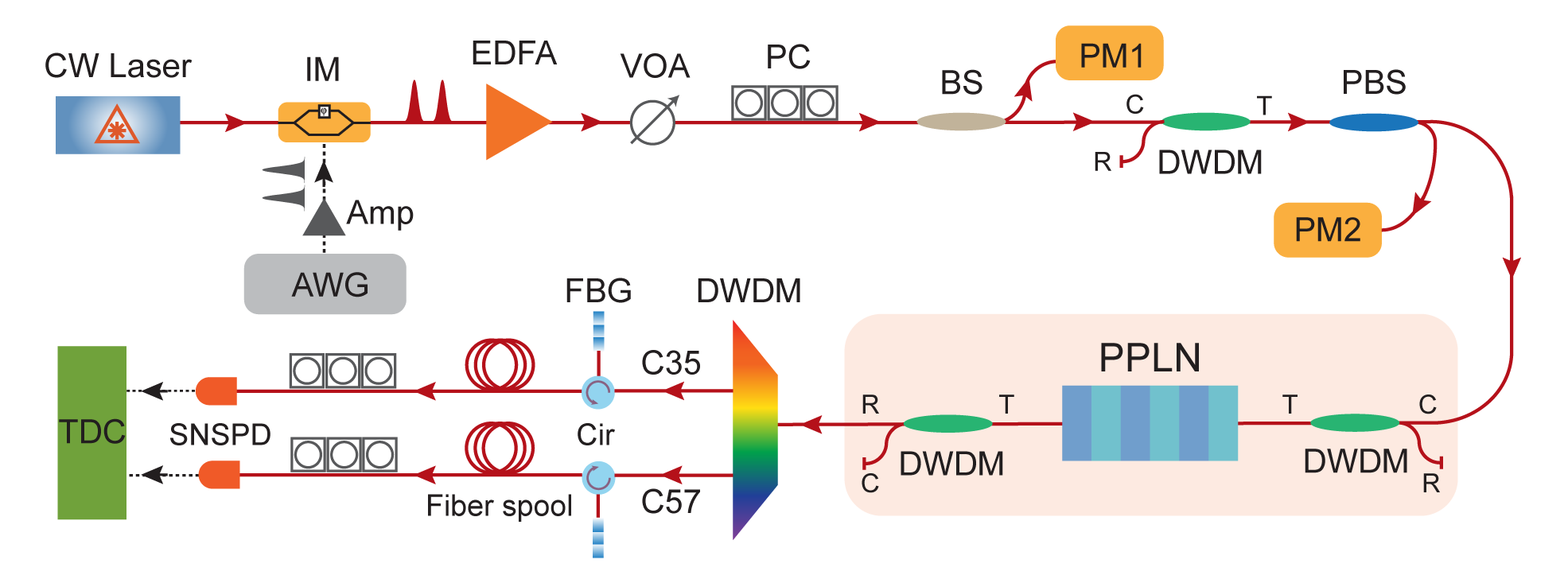
**Fig. S1. Experimental setup for generation and characterization of entangled photons pairs.** CW-Laser: continuous wave laser, IM: intensity modulator, EDFA: erbium-doped fiber amplifier, VOA: variable optical attenuator, PC: polarization controller, BS: 99:1 beam splitter, PM: powermeter, DWDM: dense wavelength division multiplexer, PBS: polarization beam splitter, PPLN: periodically poled lithium niobate, Cir: circulator, FBG: fiber Bragg grating, SNSPD: superconducting nanowire single photon detector, TDC: time-to-digital converter.


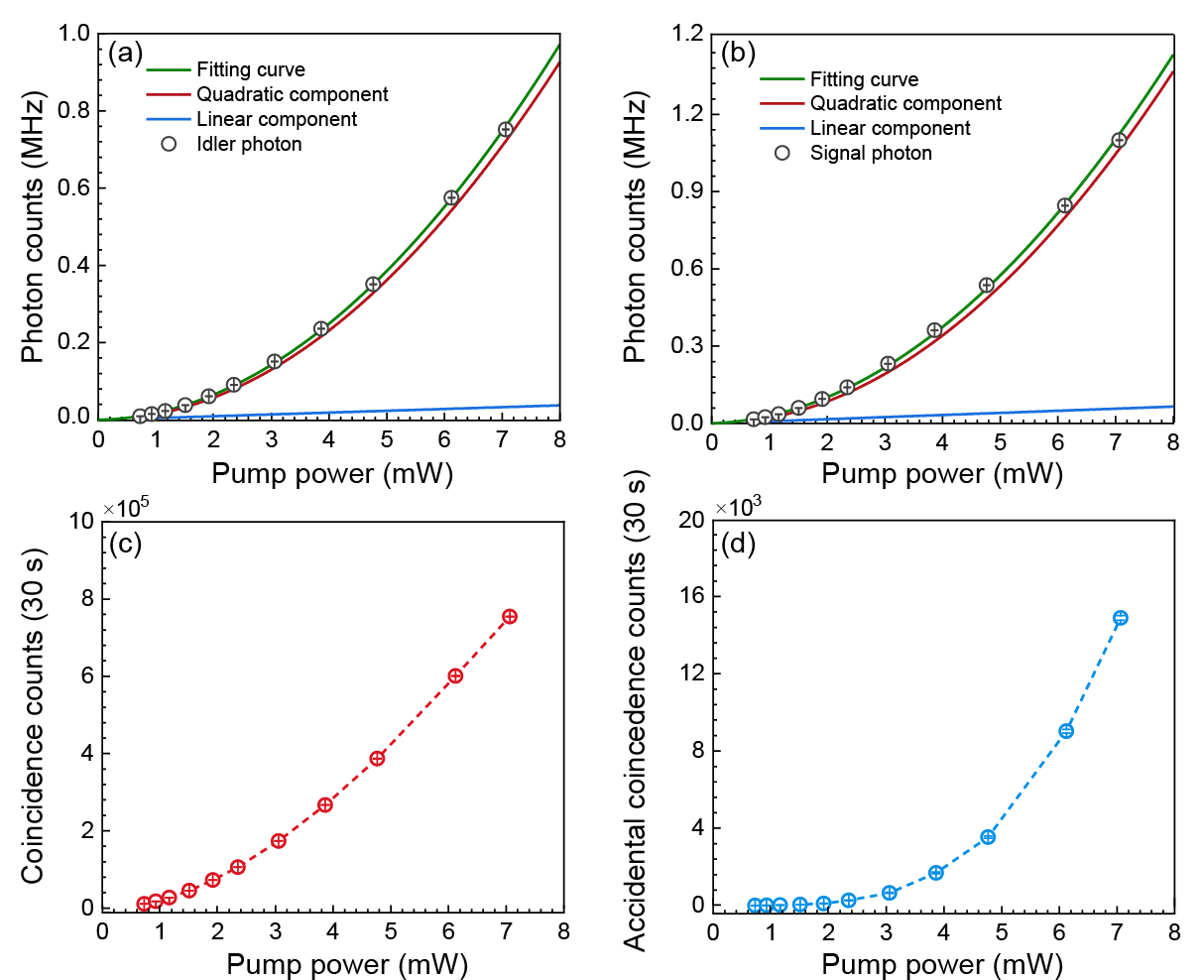
**Fig. S2. Properties of entangled photon pairs. a** and **b** Idler and signal photon counts versus pump power. The black circles represent measured idler and signal counts with different pump powers. The green line is the quadratic polynomial fitting curve of the measured counts. The quadratic and liner parts are shown as the red and blue lines, respectively. **c** and **d** Coincidence counts and accidental coincidence counts versus pump power. The red circles are measured coincidence counts within a coincidence window of 200 ps for 30 seconds under different pump power levels. The blue circles are measured accidental coincidence counts within a coincidence window of 200 ps for 30 seconds under different pump power levels.


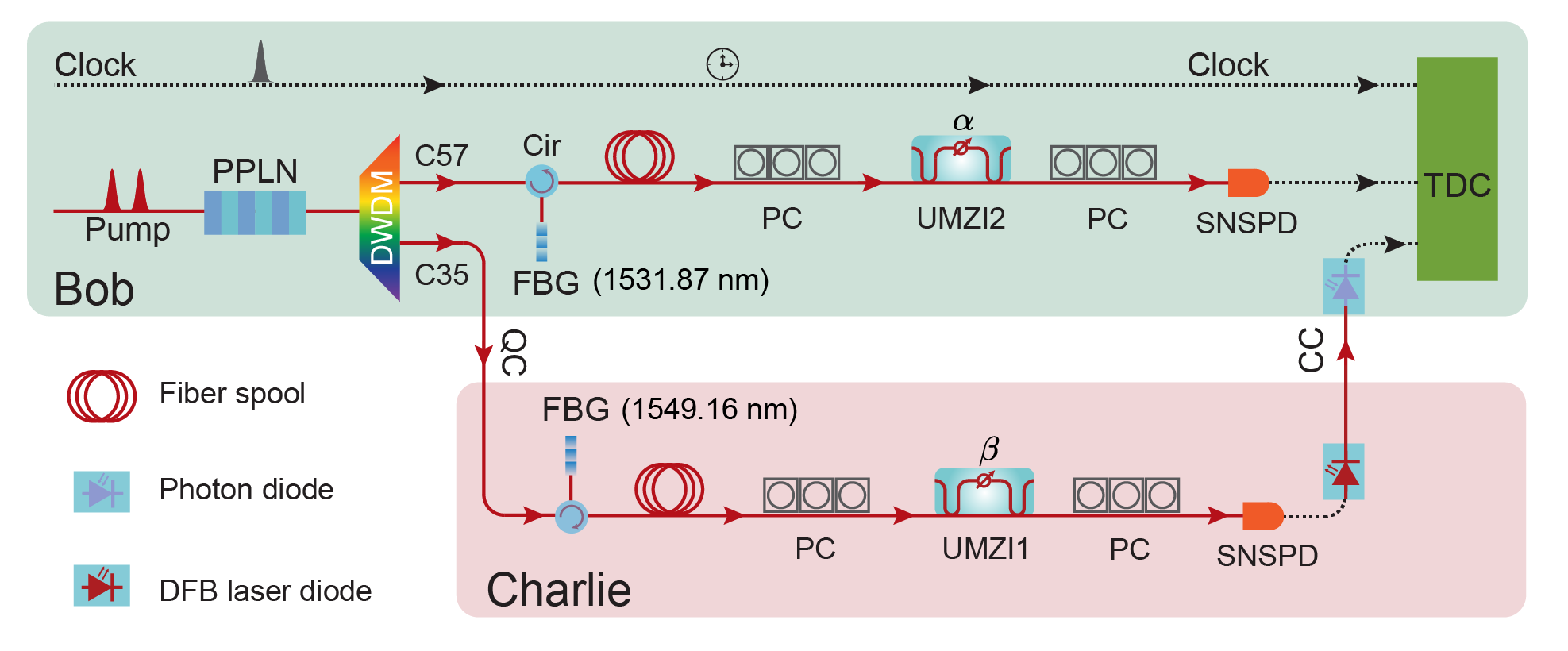
**Fig. S3. Schematic for characterization of prior entanglement distribution.** PPLN: periodically poled lithium niobate, DWDM: dense wavelength division multiplexer, Cir: circulator, FBG: fiber Bragg grating, PC: polarization controller, UMZI: unbalanced Mach-Zehnder interferometer, **S**NSPD: superconducting nanowire single photon detector, TDC: time-to-digital converter, QC: quantum channel, CC: classical channel.


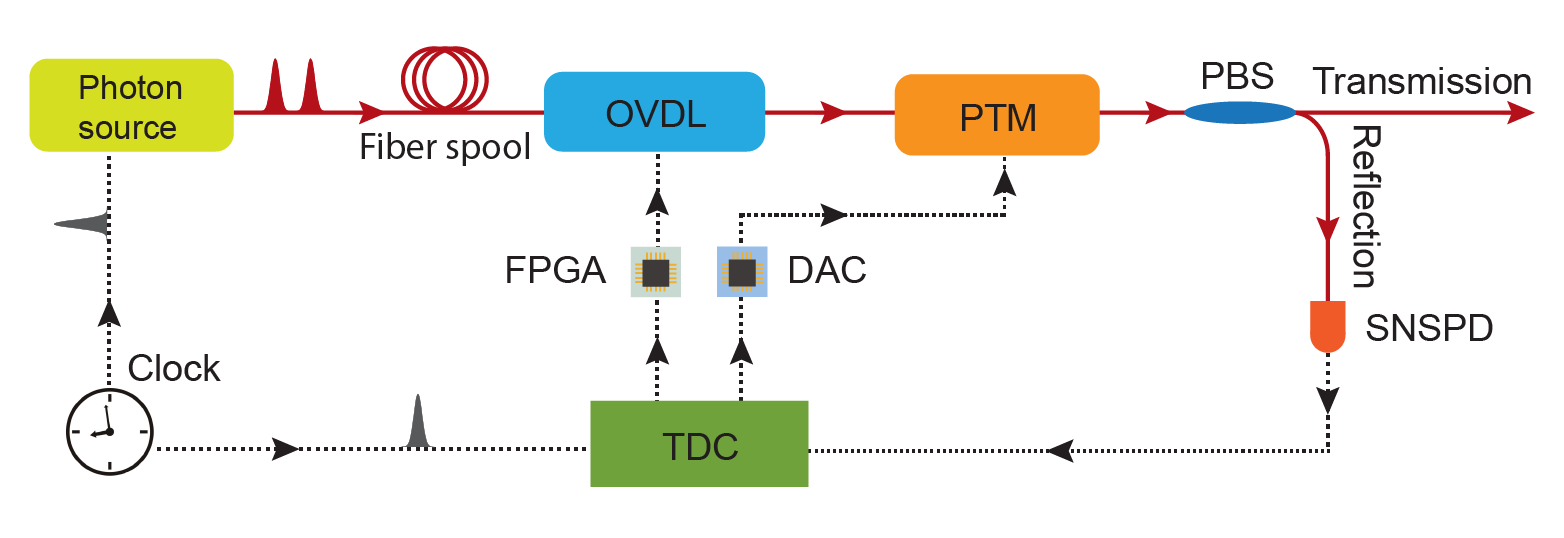
**Fig. S4. Schematic for automatic timing and polarization control.** Photon source: photons from Alice/Bob, OVDL: optical variable delay line, PTM: polarization track module, FPFA: field-programmable gate array, DAC: digital to analog convertor, PBS: polarization beam splitter, SNSPD: superconducting nanowire single photon detector, TDC: time-to-digital converter.
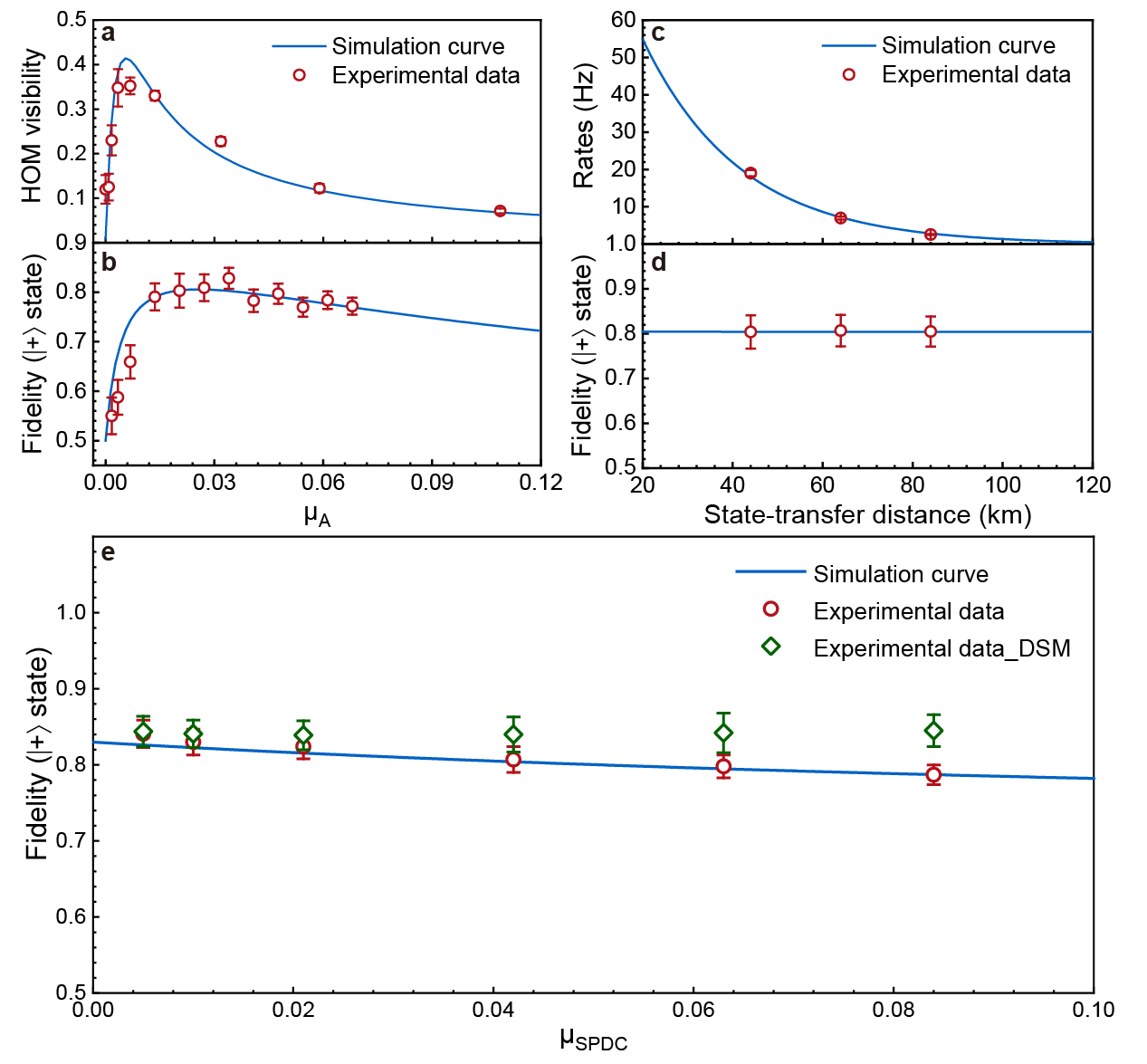
**Fig. S5. Calculated result and experimental data of HOM visibilities, teleportation fidelities of** **state and teleportation rates**. **a** and **b** Calculated and measured HOM visibilities and teleportation fidelities of state with different mean photon numbers () per qubit at Alice. For all the measurements, = 0.042. **c** and **d** Calculated and measured teleportation rates (maximum three-fold coincidence counts) and teleportation fidelities of state with different state-transfer distances. **e** Calculated and measured teleportation fidelities of state with different mean photon pair numbers. Notice that, the circles and lines in (a-e) correspond to the experimental data and calculated results, respectively.

Table S1.

Parameters of PPLN module.

| Type of waveguide | RPE waveguide |
| --- | --- |
| Length of waveguide | 50 mm |
| QPM period | 19 μm |
| SHG normalized conversion efficiency | 500%[/W@1540.56](mailto:/W@1540.56) nm |
| Length of pigtail | 20 cm |
| Input coupling efficiency of PPLN waveguide | 73.7% |
| Output coupling efficiency of PPLN waveguide | 85.7% |

Table S2.

Experimental parameters in our system.

| Parameter | Description | Result |
| --- | --- | --- |
|  | Repetition rate of qubit | 500 MHz |
|  | Average entangled photon pair number per qubit | 0.042 |
|  | Average teleported photon number per qubit | 0.029 |
|  | Transmission probability of teleported photons | 0.147 |
|  | Transmission probability of idler photons | 0.012 |
|  | Transmission probability of signal photons | 0.014 |
|  | Detection efficiency of the SNSPDs for BSM | 0.60 |
|  | Detection efficiency of the SNSPD for signal photons | 0.80 |
|  | Indistinguishability of BSM photons | 0.89±0.02 |

Table S3.

Gains [Hz] for different input states and mean photon number.

| state | signal | decoy | vacuum |
| --- | --- | --- | --- |
|  | 9.92±0.22 | 3.78±0.14 | 0.66±0.06 |
|  | 10.35±0.23 | 4.01±0.14 | 0.66±0.06 |
|  | 5.76±0.17 | 1.92±0.10 | 0.34±0.04 |
|  | 6.04±0.17 | 2.01±0.10 | 0.32±0.44 |

Table S4.

Fidelities for different input states and mean photon number.

| state | signal | decoy | vacuum | single-photon |
| --- | --- | --- | --- | --- |
|  | 92.8±0.6% | 90.3±1.1% | 53.4±4.4% | ≥97.8±1.8% |
|  | 91.9±0.6% | 89.7±1.1% | 53.0±4.4% | ≥96.6±1.7% |
|  | 76.1±1.3% | 84.9±1.9% | 56.5±6.2% | ≥89.7±3.0% |
|  | 74.1±1.3% | 81.1±2.0% | 52.4±6.3% | ≥84.9±3.0% |

Table S5.

Gains [Hz] of equatorial states for different .

|  | signal | decoy | vacuum |
| --- | --- | --- | --- |
| 0.005 | 0.70±0.03 | 0.21±0.02 | 0.02±0.01 |
| 0.010 | 1.34±0.04 | 0.46±0.02 | 0.03±0.01 |
| 0.021 | 2.42±0.04 | 0.92±0.02 | 0.07±0.01 |
| 0.042 | 5.76±0.17 | 1.92±0.10 | 0.34±0.04 |
| 0.063 | 8.90±0.21 | 3.08±0.12 | 0.66±0.06 |
| 0.084 | 12.20±0.25 | 4.42±0.15 | 1.22±0.08 |

Table S6.

Fidelities of equatorial states for different .

|  | signal | decoy | vacuum | DSM fidelity |
| --- | --- | --- | --- | --- |
| 0.005 | 80.2±1.1% | 84.1±1.8% | 53.3±15.3% | ≥84.4±2.0% |
| 0.010 | 77.6±1.1% | 83.0±1.7% | 58.3±22.3% | ≥84.1±1.8% |
| 0.021 | 77.8±1.1% | 82.4±1.6% | 59.5±8.5% | ≥83.9±1.9% |
| 0.042 | 77.8±1.0% | 80.7±1.7% | 51.4±6.0% | ≥84.0±2.3% |
| 0.063 | 75.5±0.9% | 79.8±1.5% | 54.0±4.0% | ≥84.2±2.6% |
| 0.084 | 75.7±0.8% | 78.7±1.3% | 54.8±3.2% | ≥84.5±2.1% |

References

1 Zhou, Q., Zhang, W., Cheng, J., Huang, Y. & Peng, J. Polarization-entangled Bell states generation based on birefringence in high nonlinear microstructure fiber at 1.5 μm. *Opt. Lett* **34**, 2706-2708 (2009).

2 Engin, E. *et al.* Photon pair generation in a silicon micro-ring resonator with reverse bias enhancement. *Opt. Express* **21**, 27826-27834 (2013).

3 Zhang, Z. *et al.* High-performance quantum entanglement generation via cascaded second-order nonlinear processes. *npj Quantum Inform.* **7**, 123 (2021).

4 Takesue, H. & Noguchi, Y. Implementation of quantum state tomography for time-bin entangled photon pairs. *Opt. Express* **17**, 10976-10989 (2009).

5 Ou, Z. Y. Quantum theory of fourth-order interference. *Phy. Rev. A* **37**, 1607-1619 (1988).

6 Li, X., Yang, L., Cui, L., Ou, Z. Y. & Yu, D. Observation of quantum interference between a single-photon state and a thermal state generated in optical fibers. *Opt. Express* **16**, 12505-12510 (2008).

7 James, D. F. V., Kwiat, P. G., Munro, W. J. & White, A. G. Measurement of qubits. *Phy. Rev. A* **64**, 052312 (2001).

8 Valivarthi, R. *et al.* Quantum teleportation across a metropolitan fibre network. *Nat. Photonics* **10**, 676-680 (2016).

9 Rarity, J. G., Tapster, P. R. & Loudon, R. Non-classical interference between independent sources. *J. Opt. B Quantum Semiclassical Opt.* **7**, S171-S175 (2005).

10 Wang, X.-B. Beating the Photon-Number-Splitting Attack in Practical Quantum Cryptography. *Phys. Rev. Lett.* **94**, 230503 (2005).

11 Lo, H.-K., Ma, X. & Chen, K. Decoy State Quantum Key Distribution. *Phys. Rev. Lett.* **94**, 230504 (2005).

12 Ma, X., Qi, B., Zhao, Y. & Lo, H.-K. Practical decoy state for quantum key distribution. *Phy. Rev. A* **72**, 012326 (2005).

13 Sun, Q.-C. *et al.* Quantum teleportation with independent sources and prior entanglement distribution over a network. *Nat. Photonics* **10**, 671-675 (2016).
